# Distraction and Expressive Suppression Strategies in Regulation of High- and Low-Intensity Negative Emotions

**DOI:** 10.1038/s41598-017-12983-3

**Published:** 2017-10-12

**Authors:** Ping Li, Wei Wang, Cong Fan, Chuanlin Zhu, Shuaixia Li, Zhao Zhang, Zhengyang Qi, Wenbo Luo

**Affiliations:** 1 Research Center of Brain and Cognitive Neuroscience, Liaoning Normal University, Dalian, China; 2 Laboratory of Cognition and Mental Health, Chongqing University of Arts and Sciences, Chongqing, China

## Abstract

The current study compared the effectiveness of distraction, an antecedent-focused strategy that involves diverting an individual’s attention away from affective terms, and expressive suppression, a response-focused strategy that involves inhibiting conscious emotion-expressive behavior during an emotionally aroused state, in the regulation of high- and low-intensity unpleasant stimuli, using event-related potentials (ERPs). Sixteen participants completed an emotion regulation experiment in which they passively viewed high- or low-intensity unpleasant images (view), solved a mathematical equation presented on high- or low-intensity negative images (distraction), or suppressed their emotional expression in response to high- or low-intensity unpleasant images (suppression). Their negative experiences after implementation of these strategies were rated by participants on a 1–9 scale. We mainly found that compared with expressive suppression, distraction yielded greater attenuation of the early phase of centro-parietal LPP when the participants responded to high-intensity stimuli. In the low-intensity condition, distraction, but not expressive suppression, effectively decreased the early phase of LPP. The findings suggest that expressive suppression works as early as distraction in the high-intensity condition; more importantly, distraction is superior to expressive suppression in regulating negative emotion, which is influenced by emotional intensity.

## Introduction

Regulation of emotions, especially when the emotions are maladaptive, is one of the most important abilities of human beings^1^. Various strategies can be used to regulate emotions, and the strategy can lead to distinct results in different situations^2–6^. For example, reappraisal, an antecedent-focused strategy that involves reinterpreting the meaning of emotional events in non-emotional terms^4^, has been found to be an effective way to decrease negative emotion^7^. Nevertheless, reappraisal appears to be ineffective when emotion is of high intensity or during the early regulatory stage^8^.

As reported in Gross’s process model of emotion regulation, regulation strategies can be divided into five sets (i.e., situation selection, situation modification, attentional deployment, cognitive change, and response modulation strategies) according to the time course of emotion generation^9^. Situation selection means that individuals will approach or avoid a specific situation in which an expected or unexpected emotion may be evoked. For instance, students may decide to study in the library to avoid being disturbed by a roommate who tells boring jokes. Situation modification means that a situation may be tailored to modify its emotional impact. For instance, a fearful student may decide to take part in speech presentation early and may adjust the position of the podium to attempt to feel more comfortable. Attentional deployment is utilized to select on which aspect of a situation an individual focuses. One form of attentional deployment that has received particular attention is distraction. This refers to diverting an individual’s attention away from the affective aspects of a situation^10,11^, such as when individuals going for an interview listen to music to relieve their nervousness. Cognitive change means that a person reevaluates the meaning of the situation, in a way that affects the emotions. One form of cognitive change that has received particular attention is reappraisal; for instance, by considering reading as a pastime, rather than as having a utilitarian purpose, reading would make more sense. Lastly, response modulation refers to influencing response tendencies once they have been elicited^9,12,13^. One form of response modulation is expressive suppression, in which applicants do not express their emotions in their faces during an interview, in order to impress the interviewers. Additionally, the antecedent-focused strategies (i.e., the first four strategies) occur prior to initiation of the emotional response, whereas response-focused strategies (i.e., response modulation strategies), occur after the emotional response has been initiated13. The overwhelming majority of event-related potential (ERP)^14^ and behavioral studies^13,15–20^ have found that reappraisal is more effective than expressive suppression. This provides indirect evidence that antecedent-focused strategies are generally more effective in the regulation of emotion than response-focused strategies^21,22^.

Since emotional processing and emotional regulation are influenced by many factors (e.g., intensity is a critical variable in emotion processing), additional factors should be taken into account when exploring the effectiveness of emotion regulation strategies^21,23^. Accordingly, the process-specific timing hypothesis^21,22^, which extends Gross’s process model of emotion regulation^9,13^, adds three important factors (cognitive resources, emotion intensity, and regulatory goals) to predict the effectiveness of regulation strategies. Based on the new framework, emotion-generative processes can compete with emotion-regulatory processes at both the early and late stages of information processing^21^. The efficacy of regulation strategies (e.g., distraction) that target early stages of emotion-generative processing should be relatively immune to emotional intensity, because those strategies require only a small amount of cognitive resources for modulation. In contrast, the effectiveness of regulatory strategies (e.g., reappraisal), which target the late stages of emotion-generative processing, should be largely influenced by emotional intensity, because of the need for more cognitive effort^21^. Specifically, distraction is adaptive in the short term for high and low emotional intensities, but it can be maladaptive in the long run, whereas reappraisal tends to be more adaptive in reaction to low-intensity emotional stimuli^21,23^.

Several studies have investigated the effect of emotional intensity on the strategies of distraction and reappraisal, based on the new framework. For instance, some studies have investigated individuals’ preferences for certain strategies in the face of different emotional intensities. The findings indicated that individuals tended to use reappraisal for relatively low-intensity negative images and to use distraction for relative high-intensity negative images^23,24^ even when participants were offered monetary reward to change their regulation preferences^3^. Another study, using the ERP technique, first studied the role of online processing of pictures with negative emotional intensity in predicting the choice of behavioral regulation and tested the consequences of implementing either distraction or reappraisal as the regulatory choice. Extending the behavioral findings mentioned above, increased amplitudes of the late positive potential (LPP), an ERP component that starts from 300 ms after stimulus onset and then persists for several seconds^25^, were uniquely related with subsequent distraction over reappraisal. (A reduction in LPP amplitudes is an index of regulatory success^26^. In addition, as the intensity of emotions increases, LPP amplitudes increase^27^). Moreover, implementation of a distraction rather than a reappraisal choice for regulating the response to high-intensity negative images led to greater attenuation of LPP and of self-reported arousal^28^. However, there are few studies comparing the effect of emotional intensity on the effectiveness of two different emotional regulation strategies. An electroencephalography (EEG) study explored how emotional intensity modulated two major cognitive regulatory strategies (distraction and reappraisal) at the implementation (during regulation) and pre-implementation (prior to regulation) stages, as well as their time courses. The behavioral results replicated the aforementioned behavioral findings by showing that, during the pre-regulatory stage, regulation of high-intensity negative emotion resulted in a distraction (over reappraisal) preference, while regulation of low-intensity stimuli resulted in a reappraisal (over distraction) preference. During the regulatory stage, in high-intensity regulation, but not low-intensity regulation, conditions, distraction resulted in larger modulation of the early phase of centro-parietal LPP than did reappraisal, relative to view. This indicated that, relative to view, implementation of reappraisal (but not distraction) required increased cognitive effort when subjects were viewing high-intensity negative stimuli^29^. Based on the process-specific timing hypothesis, the prior studies mentioned above suggested that emotional intensity affects the choice between distraction and reappraisal as regulatory strategies, as well as the effectiveness of these two emotion regulation strategies.

The developed framework can also be applied to other emotion regulation strategies other than distraction and reappraisal (e.g., expressive suppression, i.e., the conscious inhibition of one’s own emotionally expressive behavior while feeling emotionally aroused^4,30,31^)^21^. Expressive suppression occurs late in the emotion-generative process after emotional response tendencies have sufficiently evolved^1^. However, previous ERP studies found that expressive suppression reduced the LPP more rapidly than did reappraisal^32^ and even affected the LPP as early as distraction (during the early LPP window)^33^, when the strategy instruction was given prior to presentation of the negative picture. Accordingly, expressive suppression may affect the emotional response at an early stage, if participants are informed how to regulate their negative emotions in advance; this may be reflected by ERP results during the early LPP window. Moreover, inhibiting emotion-expressive behavior is a cognitively demanding form of regulation^31^. In contrast, according to the new framework, distraction can be expected to provide short term relief from high- (and low-) emotional intensity stimuli, with minimal effort^21^. More effort is required for modulating high-intensity emotion; thus, in conditions where resources are limited, distraction might be more efficient than expressive suppression. Emotional intensity determines the amount of regulation required; therefore, regulation strategies were generally more efficient in response to high-, as compared to low-intensity stimuli^29^. Correspondingly, compared with the high-intensity condition, suppression may be less effective or even ineffective in the low-intensity condition. These may be reflected by differences in LPP modulation, based on a prior study^29^ that found that differences in LPP modulation could reflect the effect of emotional intensity on distraction and reappraisal. Taken together, distraction may be more effective than expressive suppression at an early stage, when a negative picture is preceded by an instruction regarding strategy. Importantly, while these prior ERP studies provided evidence for emotional regulation by distraction and expressive suppression, none of these studies evaluated the important moderating role of emotional intensity, while the corresponding time courses are also still unclear.

Hence, the main goal of this study was to investigate the mechanisms underlying distraction and expressive suppression strategies for modulating high- and low-intensity negative emotion by means of high temporal resolution of ERP in an emotion regulation (ER) paradigm. In this paradigm, which is similar to the one used in a previous ERP study that examined the temporal brain dynamics of the effects of ER strategies (distraction and reappraisal) on emotional response^34^, participants were informed in advance on how to regulate their negative emotions. Based on the new conceptual framework and previous studies, we expected that distraction would be more effective than expressive suppression when negative pictures had a high intensity. Neurally, we expected that both distraction and expressive suppression would affect emotional response at the early stage. We surmised that LPP differences would be induced during the early LPP window. The distraction condition would show a larger modulation of the early phase of the centro-parietal LPP in response to high- and low-intensity negative images than would the expressive suppression condition (both relative to view). In the low-intensity condition, expressive suppression would even fail to modulate the early phase of LPP.

## Results

### Behavioral measure of negative experience

**Figure 1:**
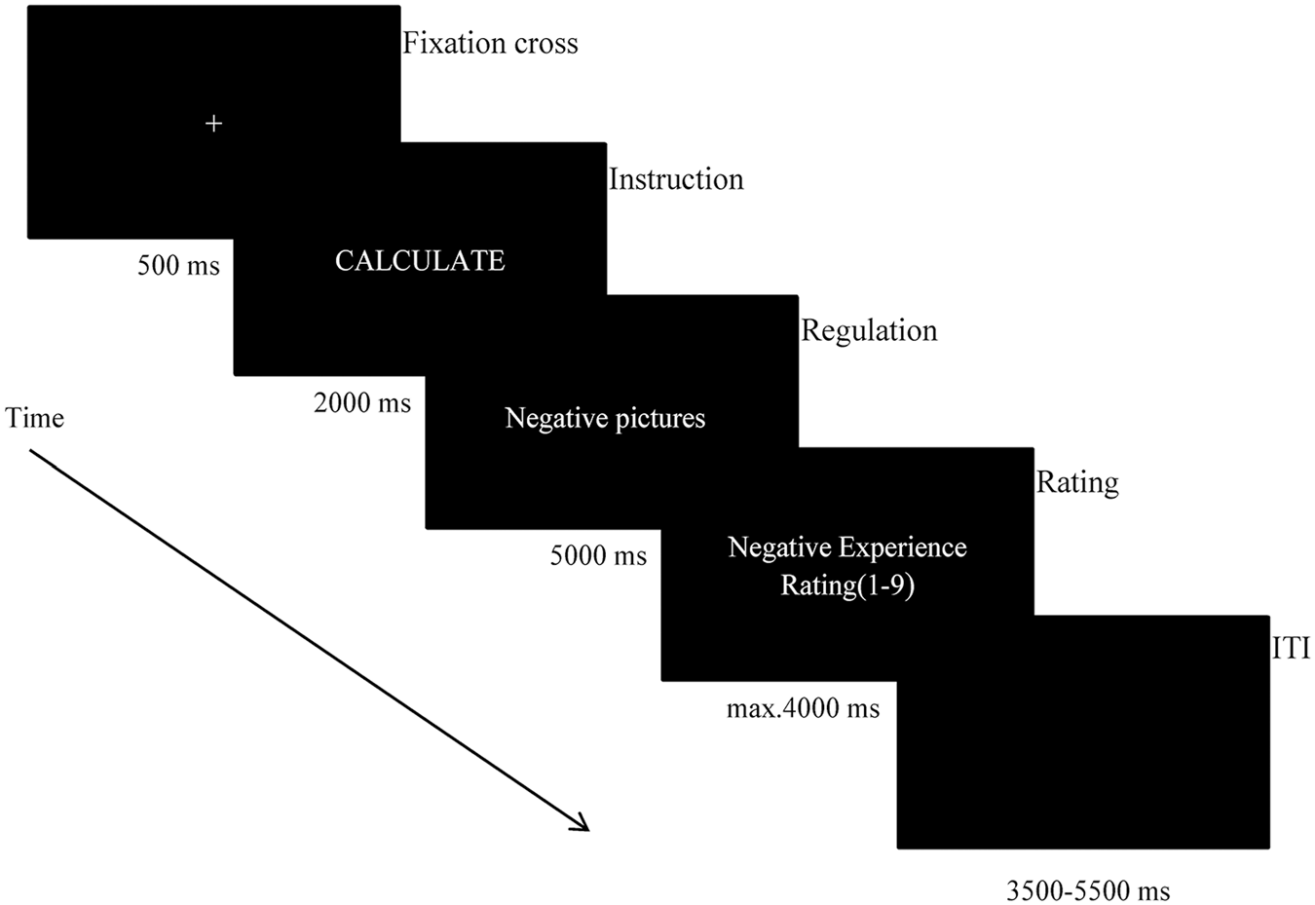
Overview of a representative experimental trial.

First, we confirmed emotional-intensity by examining whether high-intensity images (M ± SE, 5.47 ± 0.04) were felt more negative than low intensity images (3.01 ± 0.28) [*F* (1, 15) = 96.52, *p* < 0.001, *η*_p_^2^ = 0.87] in the view condition. We adopted a 2 × 3 analysis of variance (ANOVA) with Emotion-Intensity (high, low) and Regulation -Type (distraction, suppression, view) as repeated measures factors to test the effect of distraction and expressive suppression in high and low emotional intensities, as shown in negative emotion ratings. We found a significant Emotion-Intensity × Regulation-Type interaction [*F* (2, 30) = 9.98, *p* < 0.001, *η*_p_^2^ = 0.399; Fig. 1]. Follow-up comparisons revealed that in the high-intensity condition, distraction (4.18 ± 0.53, *p* = 0.01) but not expressive suppression (4.98 ± 0.48, *p* = 0.18) was efficient in reducing negative experience compared with free viewing (5.47 ± 0.39). In the low-intensity condition, we found that expressive suppression (2.74 ± 0.33, *p* = 01.15) and distraction (2.73 ± 0.35, *p* = 0.32) may not affect negative experience compared with free viewing (3.01 ± 0.28).

### ERP results

**Figure 2:**
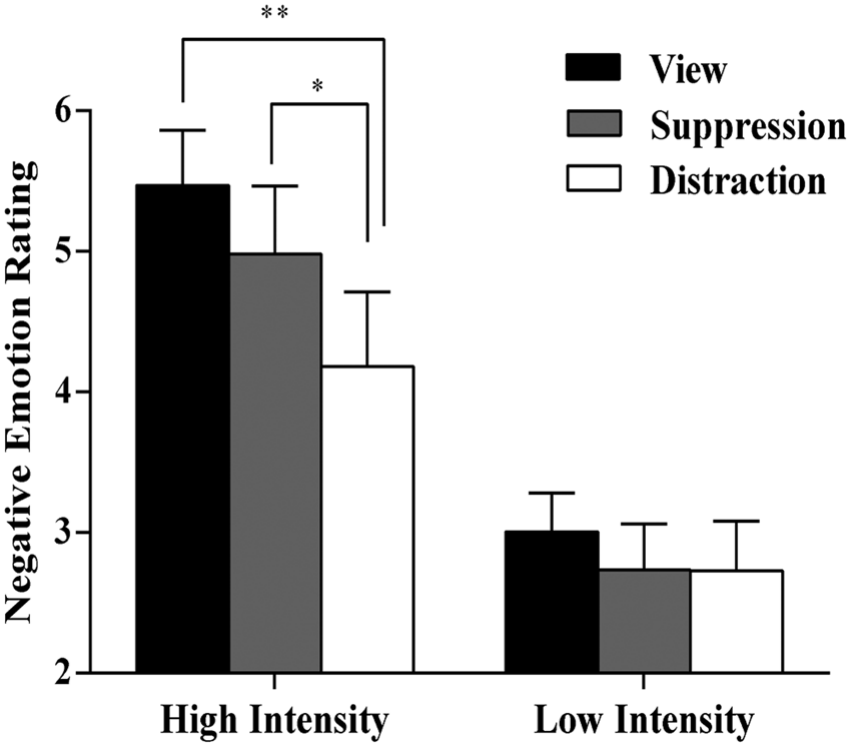
Behavioral Results: Negative Experience Ratings. Negative experience ratings for Distraction, Suppression and View at both high and low intensities. *p < 0.05; **p < 0.01.

To examine the neural regulation distinction between distraction and expressive suppression in high and low emotional intensities during early and late time windows, we employed a three-way ANOVA on LPP magnitudes with time window (early, late), emotional intensity (high, low) and strategy (distraction, suppression, view) as repeated-measures factors. The results revealed a significant interaction between time, emotional intensity and strategy [*F* (2, 30) = 4.07, *p* =0 .027, *η*_p_^2^ =0 .21; Fig. 2]. Decomposition of the significant three-way interaction indicated that in high [*F* (2, 30) = 18.57, *p* < 0.001, *η*_p_^2^ = 0.55] and low-intensity [*F* (2, 30) = 6.53, *p* = 0.004, *η*_p_^2^ = 0.30], expressive suppression and distraction had different patterns in the time window during which they have their major influences on the amplitude of LPP. Follow-up comparisons showed that, in high-intensity, strategies had different results during the early time window [*F* (2, 30) = 25.82, *p* < 0.001, *η*_p_^2^ = 0.63] but not during the late time window [*F* (2, 30) = 1.05, *p* = 0.36, *η*_p_^2^ = 0.07]. As predicted, although distraction (M ± SE, 6.45 ± 0.87 µV, *p* < 0.001) and expressive suppression (9.16 ± 1.11 µV, *p* = 0.001) were both efficient in modulating the LPP relative to view (12.13 ± 1.18 µV), distraction attenuated larger amplitudes of LPP than expressive suppression (*p* = 0.012) during the early period (300–1200 ms). During the late period (1200–2100 ms), both distraction (4.20 ± 0.60 µV, *p* = 0.77) and expressive suppression (4.19 ± 0.85 µV, *p* = 0.40) may not affect the amplitude of LPP relative to view (5.34 ± 1.02 µV). In low-intensity, there was a significant interaction between time window and strategy [*F* (2, 30) = 6.53, *p* = 0.004, *η*_p_^2^ = 0.30], which was different from the one in the high-intensity condition. Follow-up comparisons indicated that strategies had different emotional regulation effects during early time window [*F* (2,30) = 14.61, *p* < 0.001, *η*_p_^2^ = 0.49] but not during late time window [*F* (2, 30) = 0.24, *p* = 0.79, *η*_p_^2^ = 0.02]. Specifically, distraction (4.11 ± 0.40 µV, *p* = 0.002), instead of expressive suppression (6.35 ± 0.61 µV, *p* > 0.05) successfully modulated LPP relative to view (6.80 ± 0.73 µV) at an early stage (300–1200 ms). At the late stage (1200–2100), both distraction (3.62 ± 0.53 µV, *p* > 0.05), and expressive suppression (3.98 ± 0.57 µV, *p* > 0.05) may not affect the LPP amplitudes relative to view (4.24 ± 0.80 µV).

## Discussion

Although emotional intensity has a powerful impact on regulatory processes, its effect in the field of affective neuroscience has been largely ignored. Using the ERP technique, the current study compared the effectiveness of distraction and expressive suppression strategies in regulation of high- and low-intensity unpleasant stimuli in the ER paradigm. Behaviorally, distraction led to greater reduction of high- but not low-intensity negative experiences than did expressive suppression. This finding extended those of a previous study^29^, by showing that distraction was more efficient than expressive suppression in reducing a high-intensity negative experience. Additionally, distraction resulted in larger modulation of the early phase of the centro-parietal LPP in response to high-intensity negative emotion than did expressive suppression. In a low-intensity negative situation, distraction, but not expressive suppression, successfully reduced the early phase of the LPP. These findings demonstrated that distraction is superior to expressive suppression in regulating negative images during the early regulatory stage, which has different patterns for different intensities, but expressive suppression had an effect as early as did distraction in high-intensity conditions. A previous study^29^ found that in high-intensity conditions, distraction resulted in a greater reduction in negative experiences and more successfully modulated the LPP than did reappraisal. Our present study found that distraction reduced a high-intensity negative experience, and resulted in larger modulation of LPP in both high- and low-intensity conditions than did expressive suppression. These findings indicate that emotional intensity has an impact on the time course of regulation, and verified that distraction is a relatively effective strategy.

The current finding that, in low-intensity conditions, distraction reduced the LPP to a marked extent, was in contrast with the findings of a prior study^29^, which reported that distraction resulted in only marginally significant reduction of LPP. This might be because the procedures of the two studies were different. That is, in our study, participants were only given a strategy instruction before they implemented the strategy, whereas in the previous study, the strategy instruction and intensity of the subsequently presented image were displayed before the participants performed emotion regulation. That is, the clue to the intensity of the image in the previous study might have reduced the motivation to perform an emotion regulation task, and thus the regulatory force of distraction was weakened.

More importantly, the ERP results in this study showed that distraction led to a larger modulation of the early phase of the centro-parietal LPP in response to high-intensity unpleasant stimuli than did expressive suppression (both compared with view). During distraction, resolution of conflict via the early filtering mechanism requires few cognitive resources^21^. In contrast, inhibition of one’s own emotionally expressive behavior requires marked effort^31^. More effort is required for modulation of high-intensity emotion; thus, when resources are limited, distraction might be more efficient than expressive suppression. Furthermore, we found that expressive suppression resulted in significant ERP changes in high-intensity but not in low-intensity conditions. This demonstrates that expressive suppression was more efficient in response to high- than to low-intensity stimuli, possibly because high-intensity stimuli require a greater amount of regulation^35^.

In terms of the regulation stage, distraction showed successful modulation, as assessed from LPP changes, during the early regulation stage. This finding replicates prior work showing that distraction began to modulate the LPP in response to negative stimuli from the early stage (at 300 ms)^36^. More importantly, we observed that expressive suppression decreased the LPP as early as did distraction when high-intensity unpleasant images were being viewed. This finding is consistent with a previous study^33^, in which the negative stimuli were of high intensity and the regulation instructions were given before image presentation. This suggests that strategy instructions given before image presentation activated the expressive suppression regulation process early on; thus, participants prepared in advance to prevent the generation of an emotional response^33^.

In terms of brain mechanisms that underly emotion regulation and affect the LPP, a previous study had revealed that a critical amygdala−prefrontal emotion-regulatory network selectively impacts behavior^37^. We agree with another study^38^, which found that changes in amygdala connections are usually compared across broad domains, such as emotion processing and attention. These results suggest that structure−function relationships between brain and emotion should be reconceptualized from a dynamic perspective.

There are two limitations in the present study. First, our study only measured the effect of emotional intensity on the effectiveness of the two strategies (distraction, expressive suppression), but how the emotional intensity influences the participants’ selection of regulation strategy is unclear. Thus, in a future study, we will address this issue by letting participants choose strategies for themselves. Second, how emotional intensity influences the effectiveness of strategies for modulating positive stimuli is unknown. Based on the developed framework, emotional intensity is one of the factors that influences the effectiveness of a regulatory strategy^21^. Therefore, emotional intensity in the context of positive images is also likely to affect the effectiveness of regulation strategies. However, in our study, participants were only shown negative-valence images. Moreover, prior studies have rarely focused on the effect of emotional intensity on emotion regulation by using positive images. Accordingly, we will explore this issue in future. Furthermore, as strategies play crucial roles in regulating negative emotions in individuals with mental disorders, such as social anxiety disorder^39,40^, depression^41^, bipolar disorder^42^, and autism^43^, we may explore whether the effect of strategy on mental illness would differ with emotion intensity in a future study.

## Conclusion

Our findings extended those of previous studies by demonstrating that distraction is superior to expressive suppression in regulating negative emotion during the early regulatory stage, which is affected by emotional intensity, and that expressive suppression works as early as does distraction in high-intensity conditions. These were reflected by the findings that distraction led to a larger modulation of the early phase of the centro-parietal LPP in response to high-intensity negative emotion than did expressive suppression. In a low-intensity negative emotion condition, distraction, but not suppression, successfully reduced the early phase of LPP. These LPP changes were induced during the early LPP window. Our study also extended previous investigations regarding the modulatory role of emotional intensity on emotion regulation in affective neuroscience. Furthermore, our results may lay the foundation for researchers and practitioners to determine effective emotion regulation strategies for improving the negative experiences of patients with neurological disorders.

## Materials and Methods

### Participants

Sixteen healthy undergraduates (8 females) ranging between 18–22-year-old (mean age = 20.1 years) took part in this study. All participants were right-handed, without psychiatric disorders history, and had normal or corrected-to-normal vision. Participants were paid a certain amount of money for their involvement after the test. Written informed consent was obtained before the experiment. The study was approved by the Ethics Committee of Chongqing University of Arts and Sciences in accordance with the Declaration of Helsinki (1991).

### Materials

A total of 80 pictures were selected from the International Affective Pictures System^44^. Consistent with previous studies^3,23,29^, we divided these pictures into high- and low-intensity on the basis of their normative ratings for arousal and valence, half of which are high-intensity negative pictures (M_arousal_ = 6.42, M_valence_ = 2.17), the other are low-intensity negative ones (M_arousal_ = 4.69, M_valence_ = 3.50). They were significantly different in valence [*t* (39) = −9.05, *p* < 0.001] and arousal normative ratings [*t* (39) = 15.477, *p* < 0.001]. Studies have established that arousal and valence differences of the magnitude separating our low intensity and high-intensity stimuli are adequate to produce different levels of emotional-response activation, as revealed by physiological arousal^45^ and electrocortical markers of negativity^46^. Picture contents included scenes of illness, accident, pain, pollutant and mutilation, and were matched for the high- and low-intensity categories.

In addition, arithmetic equations that were used in the distraction trials included one addition and one subtraction (e.g., 6 + 7 − 5 = 8). Participants were asked to calculate the equations and then press the button as quickly and accurately as possible. Half of the equations are incorrect and the incorrect answer was different by 1 from the correct one. 140 arithmetic problems were tested in an independent sample of 20 healthy participants. From these, 80 equations were screened in order that all of them took on average more than 2000 ms to be calculated. Then, the 80 equations were randomly assigned to the background picture category (high intensity, low intensity) in the distraction condition so that there were no significant differences in reaction times [overall mean: 3197 ms; *t* (39) = −0.76, *p* = 0.45].

### Procedure

The paradigm was similar to the ERP study conducted by Schönfelder *et al*.^34^. An instruction cue (the words “VIEW”, “CALCULATE” and “SUPPRESSION”) appeared before a picture presentation, which signaled the subsequent emotion regulation strategy. Additionally, three corresponding tasks were included. During distraction task (CALCULATE), participants were asked to ignore the background picture by solving the presented mathematical equation and judging whether the displayed solution was correct or not. During suppression task (SUPPRESSION), participants’ responses to the unpleasant pictures were suppressed and their expressive behaviors were inhibited in order to avoid being found by other people (e.g., putting on a poker face when playing cards). During passive viewing task (VIEW), participants watched the picture seriously and responded naturally without changing their emotional responses. Before the formal experiment, participants were instructed to learn how to carry out the two strategies and practice both for 8 trials. To ensure the participants had understood the instructions, they also had to explain the emotion regulation instructions to the experimenter. Moreover, in the formal experiment, the participants were asked how they employed two strategies after every block. Participants were corrected by the experimenter as needed. This procedure revealed that all participants were able to interpret the implementations of both strategies.

**Figure 3:**
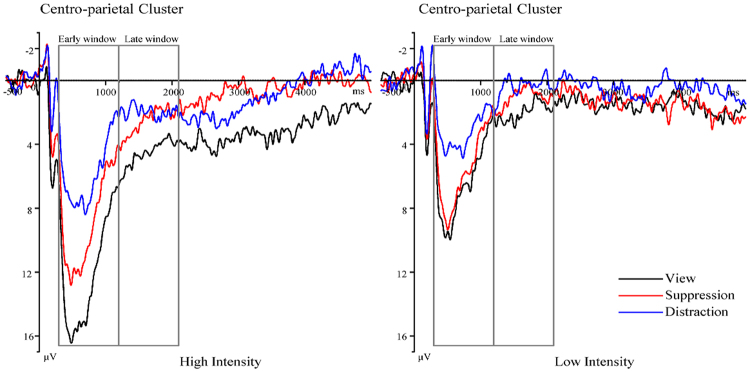
Picture-locked LPP amplitudes for Distraction (blue lines), Suppression (red lines) and View (black lines) at high and low intensities. ERP activity was averaged across centro-parietal electrodes (Pz, CPz).

In the formal experiment, trial sequence was as follows (see Fig. 3). First, a fixation cross (500 ms) was presented to make participants concentrate on the screen. This was followed by an instruction cue (2000 ms) which indicated how to regulate their negative emotion once they saw a negative picture. Next, an emotional picture was presented for 5000 ms, during which participants performed one of three tasks (passive viewing, distraction and suppression) according to the practiced strategies. At the end of each trial, participants needed to rate their current negative experience on a 9-point scale (1 = not negative at all, 9 = extremely negative)^44^.

The procedure included five blocks and each one had 48 trials. Of the 240 trials, high-intensity and low-intensity negative pictures were equally and randomly presented with no more than two consecutive trials of the same intensity. And these pictures were randomly combined with three emotional regulation types that were equivalently presented (Distraction, Suppression, View), making each experimental condition involve 40 trials.

### Data Recording and Analysis

Brain electrical activity was recorded by Vision Recorder software (Brain Product) using a 64-channel amplifier, based on the extended International 10–20 system. All recordings were online referenced to the left mastoids, and re-referenced offline to the average of the left and right mastoids. Horizontal electrooculographies (EOGs) were recorded from two electrodes sites at the outer canthi of each eye and vertical EOGs were measured from tin electrodes located at infraorbital and supra-orbital regions of the right eye. EEG and EOG signals were sampled at a rate of 500 Hz and band-pass filtered from 0.01 Hz to 100 Hz. All electrode impedances were kept below 5 kΩ.

The EEG data were analyzed offline with Analyzer 2.0 software (Brain Products). We analyzed ERPs evoked by negative emotional pictures. Continuous EEG data were band-pass filtered with a 0.1 to 30-Hz (24 dB/oct). An independent component analysis algorithm was applied to correct eye-movement artifacts. EEG epochs were subsequently extracted using a wider time window of 5500 ms (500 ms pre-stimulus and 5000 ms post-stimulus). EEG and EOG artifacts were eliminated when the amplitude of any electrode exceeded ± 100 µV. Approximately 12.5% of the trials were rejected from the average. The remaining trails (more than 34 trails per condition) were used for averaging EEGs. Based on grand-mean ERP topographies, Pz and CPz, which were also the most consistently reported for analyzing the centro-parietal LPP in prior studies^25,29^, were used for the analysis of this ERP component in our study. Based on grand-mean ERP topographies, the centro-parietal LPP was measured as the average activity of Pz and CPz in our study, which were also the most consistently reported for analyzing this ERP component in prior studies.

We divided the centro-parietal LPP period into early and late time windows according to visual detection of grand-mean waveforms and previous studies^29,33^. The early window was from 300 ms to 1200 ms post-picture onset. The late time window began at 1200 ms and was of the same length as the former one. Early vs. late time windows in the neural or cognitive processing domain are specific time courses in ERP. The LPP amplitudes were averaged over the early and late time windows. The *p*-values were corrected by the Greenhouse-Geisser correction.
